# Exploring Cancer Incidence, Risk Factors, and Mortality in the Lleida Region: Interactive, Open-source R Shiny Application for Cancer Data Analysis

**DOI:** 10.2196/44695

**Published:** 2023-04-20

**Authors:** Didac Florensa, Jordi Mateo-Fornes, Sergi Lopez Sorribes, Anna Torres Tuca, Francesc Solsona, Pere Godoy

**Affiliations:** 1 Department of Computer Engineering University of Lleida Lleida Spain; 2 Population-based Cancer Registry Santa Maria University Hospital Lleida Spain; 3 Field Epidemiology Unit Lleida Biomedical Research Institute Lleida Spain; 4 CIBER Epidemiology and Public Health (CIBERESP) Health Institute Carlos III Madrid Spain

**Keywords:** R Shiny, cloud computing, microservices, Docker, decision support system, cancer incidence, cancer risk factors, cancer mortality

## Abstract

**Background:**

The cancer incidence rate is essential to public health surveillance. The analysis of this information allows authorities to know the cancer situation in their regions, especially to determine cancer patterns, monitor cancer trends, and help prioritize the allocation of health resource.

**Objective:**

This study aimed to present the design and implementation of an R Shiny application to assist cancer registries conduct rapid descriptive and predictive analytics in a user-friendly, intuitive, portable, and scalable way. Moreover, we wanted to describe the design and implementation road map to inspire other population registries to exploit their data sets and develop similar tools and models.

**Methods:**

The first step was to consolidate the data into the population registry cancer database. These data were cross validated by ASEDAT software, checked later, and reviewed by experts. Next, we developed an online tool to visualize the data and generate reports to assist decision-making under the R Shiny framework. Currently, the application can generate descriptive analytics using population variables, such as age, sex, and cancer type; cancer incidence in region-level geographical heat maps; line plots to visualize temporal trends; and typical risk factor plots. The application also showed descriptive plots about cancer mortality in the Lleida region. This web platform was built as a microservices cloud platform. The web back end consists of an application programming interface and a database, which NodeJS and MongoDB have implemented. All these parts were encapsulated and deployed by Docker and Docker Compose.

**Results:**

The results provide a successful case study in which the tool was applied to the cancer registry of the Lleida region. The study illustrates how researchers and cancer registries can use the application to analyze cancer databases. Furthermore, the results highlight the analytics related to risk factors, second tumors, and cancer mortality. The application shows the incidence and evolution of each cancer during a specific period for gender, age groups, and cancer location, among other functionalities. The risk factors view permitted us to detect that approximately 60% of cancer patients were diagnosed with excess weight at diagnosis. Regarding mortality, the application showed that lung cancer registered the highest number of deaths for both genders. Breast cancer was the lethal cancer in women. Finally, a customization guide was included as a result of this implementation to deploy the architecture presented.

**Conclusions:**

This paper aimed to document a successful methodology for exploiting the data in population cancer registries and propose guidelines for other similar records to develop similar tools. We intend to inspire other entities to build an application that can help decision-making and make data more accessible and transparent for the community of users.

## Introduction

Cancer morbidity and mortality are increasing worldwide despite the development of new prevention strategies and screening programs. This increase can be attributed to several factors, including population growth, aging, and changes in lifestyle and environmental factors. The authors of [1] estimated that the global number of cancer patients (incidence rate) will increase over the coming years due to negative lifestyle and demographic changes related to population aging and growth.

The cancer incidence rate is essential for public health surveillance [2]. The incidence rate approximates the average risk of developing cancer, allowing geographic comparisons of the disease risk in different populations. This calculation requires a population-based cancer registry (PBCR) to record, store, and organize all the cancer cases in a reference region. This is achieved by a continuous process of systematic collection, storage, analysis, interpretation, and reporting of data on the occurrence and characteristics of cancer cases [3].

Over recent decades, there has been an exponential growth in PBCRs. The first volume of the Cancer Incidence in Five Continents (CI5), published in 1966, contained information from 32 registries in 29 countries, whereas the latest volume, published in 2021, included information from 343 PBCR in 65 countries.

Several data sources are integrated into PBCRs, including hospitals, death certificates, and laboratory services. Moreover, PBCRs follow international procedures, ensuring high-quality and reliable data. These goals are accomplished by performing exhaustive (automatic and manual) validity checks [4].

PBCRs are commonly used in epidemiological research. Thus, they have a crucial role in providing extensive information about tumor histology, stage at diagnosis, place and nature of the treatment, and survival [5]. Descriptive studies use registry databases to examine differences in incidence, survival, and prevalence of risk factors or comorbidities (obesity, tobacco consumption, or diabetes) across populations and their context (such as variables associated with time, place, sex, ethnicity, and social status) [6,7].

The data sets and databases stored in PBCRs grow year on year. Data visualization is essential for exploring and communicating findings in medical research, especially in epidemiological surveillance. Hence, there is an intrinsic need for rapid raw data visualization. The current situation and context (historical data) can be understood by navigating among descriptive analyses, and, before executing time-consuming predictive or prescriptive models, it is essential to generate alarms and accurate predictions or discover hidden trends or patterns.

Previous literature has described the research of the implementation of web platforms to analyze data information related to cancer. Petrov and Alexeyenko [8] implemented an application to explore molecular features and responses to anticancer drugs. Deng et al [9] presented another web application implemented on R Shiny that permitted the analysis of molecular cancer gene data sets. The user can analyze outcomes from individual genes and cancer entities. A similar application was designed by Yang et al [10]*.* It also analyzed and provided information on cancer gene isoform expression. Finally, another application about cancer genes was presented by Dwivedi et al [11]. In this case, it was used to perform a survival analysis on single-cell RNA sequencing data. A study by van de Water et al [12] presented a web-based tool to inform patients about esophagogastric cancer treatment options and their outcomes. These kinds of web applications can also be linked to a trained prediction tool, as demonstrated by Xu et al [13]. They developed a sexually transmitted infection prediction tool. Therefore, the literature has focused on cancer genes, cancer treatments, or other diseases, but few applications are based on epidemiological cancer data. In addition, our system is entirely adaptable to other PBCRs.

Currently, PBCRs expend resources and time to extract, analyze, and present the data to gain insight into the incidence, mortality, and survival rates for cancer. Moreover, these insights are generated manually.

One approach to solving this limitation is to develop a generic platform based on microservices for PBCRs capable of generating interactive plots, tables, and statistics to determine the epidemiological cancer situation. To address this challenge, in this paper, we propose a platform capable of (1) navigation across time and feature-based data, (2) plotting aggregated and disaggregated data on demand, and (3) automatic integration of new data.

The core activities of the PBCR have expanded beyond the provision of data to perform epidemiological research or the provision of cancer reports and statistics for a region. The data in PBCRs are the basis for estimating the cancer burden and its trends over time and are crucial in the scheduling and evaluation of cancer control programs in the registration area. One of the simplest ways of tackling this problem is to use segregated information to convince authorities about which population segments need more or different attention. For instance, geographical heat maps can be used to spot differences across urban or rural areas, while age pyramids can highlight age group differences. This can help authorities to invest and generate personalized prevention campaigns.

In summary, in this article, we propose a seed to develop this platform. The main contributions are the presentation of a successful case study for Lleida PBCR and guidelines to evolve these into a reference that can be adopted by the community. The platform was designed to be differentiated by end user. One end user is the PBCR professional who analyzes the incidence of cancer in a specific region and makes decisions to research or prevent cancer. Another end user is the nonprofessional user who wants to know the cancer situation in his or her area.

The paper is structured as follows. The next section presents the methodology involved in designing and implementing the web platform. The Results section describes the different views implemented in this application and how the customization works. The presented data visualizations are related to cancer incidence, risk factors, and mortality. Finally, the results are discussed in the Discussion section, which also includes our conclusions.

## Methods

The application is based on the model-view-controller pattern. For the visual part, we used the open-source programming language R [14] in conjunction with RStudio [15], an open-source integrated desktop environment for R. The database was created by MongoDB [16], an open-source, nonrelational database, and based on document store database, where documents are grouped into collections according to their structure. To communicate these systems and obtain the information, we implemented an application programming interface (API). Finally, to encapsulate this system and facilitate the deployment, we ran it into Docker containers that Docker Compose orchestrated [17]. Docker permits encapsulating and deploying the execution of applications in packages. All these technologies are free of charge. The deployment and code are available to download in this GitHub repository [18].

### Workflow

Until the implementation of this application, PBCR professionals were manually extracting the data on demand. Once the cases were received, they cleaned and prepared the tables and plots to analyze them. Finally, they added these results to a formal report sent to public health officials.

However, once the application has been deployed, the professionals can automatically present the data to public health officials. The data extraction and cleaning steps are done by an extract, transform, and load system deployed in a server; therefore, they do not need to spend time preparing the data. In addition, the application permits real-time comparison of cancer cases between the previous years. The following subsections show how the web application has been designed and implemented.

### Front-end Service

The front end was implemented using the Shiny [19] package from the R programming language, making it easy to build interactive web applications. Shiny allows R users to create interactive web applications without extensive knowledge of web design. It also permits standalone applications to be hosted on a web page and extends the application with CSS themes, html widgets, and Javascript actions.

All the plots were made using the plotly library [20], which is defined as an interactive, open-source, browser-based graphing library. It contains over 30 types of plots, including scientific charts, statistical charts, 3D graphs, and more. The tables were made using DataTable [21], defined as a plug-in for the jQuery Javascript library, which enabled the building of interactive and flexible tables. The map was made with the GeoJSON package [22]. It is a format for encoding a variety of geographic data structures and uses a geographic coordinate reference system. It also permits a specific zone and highlighted part of this map to be represented by a palette of colors.

### Back-end Service

The back end consisted of an API and a database for the web application. Both these services were encapsulated using the Docker system, which permits scalability to other infrastructures. The API established the communication between the database and the view. This system was implemented by NodeJS [23], which can be described as an open-source environment based on the JavaScript programming language. This technology has increased exponentially over the last few years because it is based on asynchronous tasks, which permit executing calls without the need to wait for a response from the previous one. In addition, this uses a single threaded model with an event loop and is based on JSON format. The database implementation was based on a nonrelational database using the MongoDB system [16,24]. It saves the information through documents that are grouped into collections. This database permits large volumes of constantly changing structured, semistructured, and unstructured data. Nonrelational databases are designed by dynamic schemes to insert data without a specific structure as the relational databases specify. Therefore, it makes it easy to make significant changes to applications in real time without service interruptions.

### Docker and Docker Compose

The front-end and back-end technologies were encapsulated into Docker containers. Docker is a platform designed to build, share, and run modern applications into containers [17] where the applications are virtualized and executed. The main purpose of these containers is to implement some processes and applications separately to take advantage of the infrastructure simultaneously. The way Docker is designed is to give a quick and lightweight environment where code can run efficiently. Docker contains 4 main internal components: Docker client and server, Docker images, Docker registries, and Docker containers [25].

These containers were defined using Docker Compose, which orchestrated all of them. It composes a set of components, each of which is an image and a set of options that specify what the component should have. It uses a configuration file where the user selects the parameters, and when it is executed, it runs the needed processes to build the Docker container. The user can reuse the same image for different components, and these images will be managed in other containers once instantiated [26].

### Data

The case data were extracted from the official Cancer Population Registry in Lleida and the Mortality Registry of Catalonia. Experts from the cancer registry previously validated these cases to ensure the validity of the tumor. In the case of mortality, the included individuals were those patients who died from cancer in the Lleida region. The cancer patients were complemented with their risk factors, extracted from the clinical history records at the time of diagnosis. This information permitted us to build the databases and show them in the visual part.

The database was structured into 3 collections: Patients, Tumors, and Mortality. The Patients collection included sociodemographic information and risk factors; the Tumors collection included such information as the diagnosis and the kind of tumor. Finally, the Mortality collection registered sociodemographic information and cause of death (tumor list). Table 1 specifies the variables in each collection.

**Table 1:** Database collections and their variables.

Variables		Specification
**Patients**		
	sex	Gender (man/woman)
	data_naix	Date of birth (date)
	postal_code	Postal code of city residence (number)
	postal_desc	Name of city residence (characters)
	comarca	Specific region in Lleida (characters)
	comarca_desc	Specific region description in Lleida (characters)
	alcoholism	Alcohol consumption (yes/no)
	diabetes	Diabetes diagnosed (yes/no)
	smoking	Smoking consumption (yes/no)
	bmi	Body mass index (number)
**Tumors**		
	data_inc_pobl	Diagnoses date (date)
	ltum	Tumor location (characters)
	ltum_desc	Tumor location description (characters)
	morf	Tumor morphology (characters)
	morf_descr	Tumor morphology description (characters)
	metode_dx	Diagnostic method (number)
	metode_dx_descr	Diagnostic method description (characters)
**Mortality**		
	data_naix	Date of birth (date)
	data_def	Date of death (date)
	cause10	Death cause (characters)
	cause10_desc	Death cause description (characters)
	sex	Gender (man/woman)
	comarca	Specific region in Lleida (characters)
	comarca_desc	Specific region description in Lleida (characters)
	yeard	Year of death (number)

### Ethical Considerations

All data were anonymized to protect patient privacy and confidentiality. The study was part of the public health response to the impact of cancer on the society. It was approved by the Committee of Ethics and Clinical Research of Lleida (CEIC 21/190-P). As it was a retrospective cohort study and the patients were blinded to the investigators, no written informed consent was necessary according to the CEIC. All methods were carried out in accordance with relevant guidelines and regulations.

## Results

This web application consisted of an intuitive analytical web platform for rapid analysis of the population cancer registry data set, containing incidence, mortality, and risk factors related to tumor information. The application shows the incidence and evolution of each cancer during a specific period for gender and age groups. It also permits knowledge of the situation of all the cancers in a particular period and subregion in Lleida. The application also summarizes patients’ risk factors detected in the cancer registry and shows results about cancer mortality. These plots enable the number of cases to be analyzed for each year, filtered by tumor location, gender, and age group.

### Cancer Incidence

**Figure 1:**
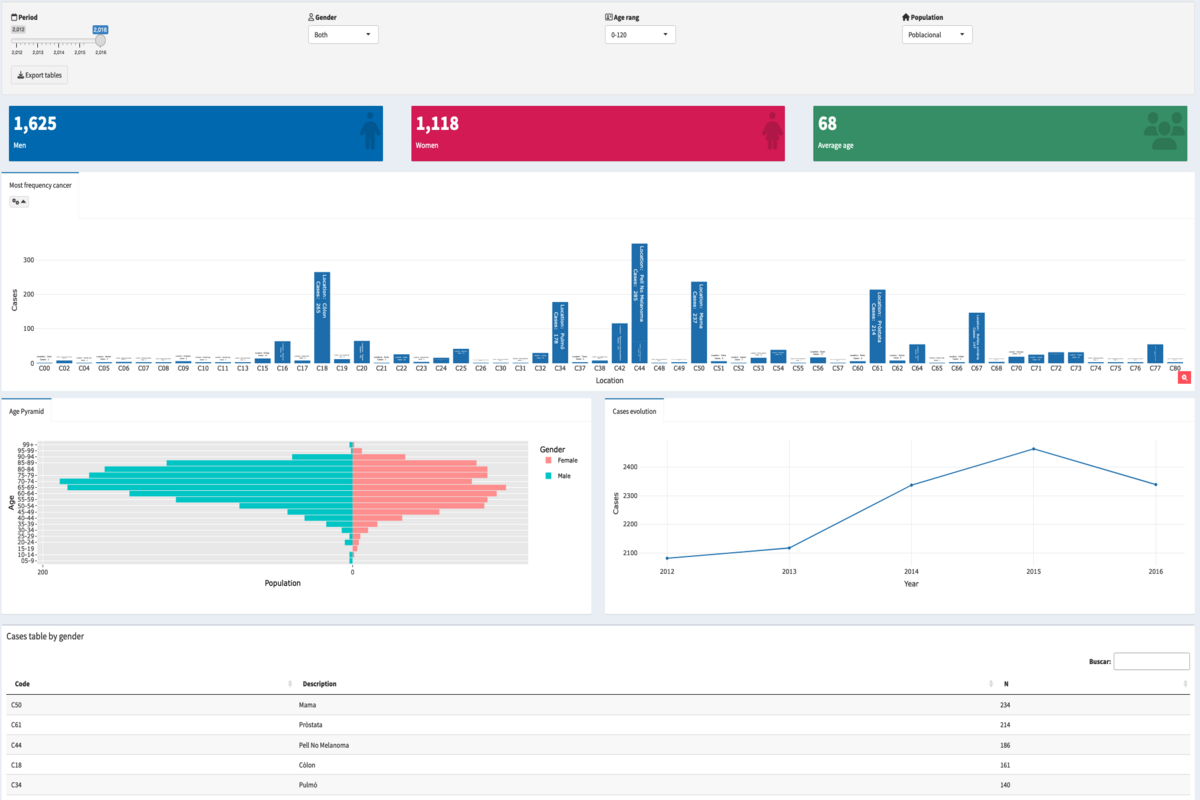
Main menu of the web application.

The web application was designed as a web browser–based dashboard (see Figure 1) to show the information according to what the user specifies in the filters. The users can filter by years between 2012 and 2016, gender, age group, and population. This last filter can show only residents of Lleida or all cases diagnosed in the reference hospitals. Below the input filters, 3 boxes show the numbers of men and women and the average age of the patients. If the user decides to filter by men, the women box will be hidden, and the average age box will be calculated only for men. Next, the bar plot represents the number of cases diagnosed by the tumor location. The pyramid age plot helps the user analyze which age group registered the most diagnosed cases among men and women. These plots can be recalculated for all the filter inputs. Next to the pyramid age plot, the display shows the evolution of the incidence for the available years, and it allows analysis of the change in men, women, or a specific age group, depending on the chosen filters. At the end, a table with the number of diagnosed cases by tumor location is displayed and can be updated using all the filters.

**Figure 2:**
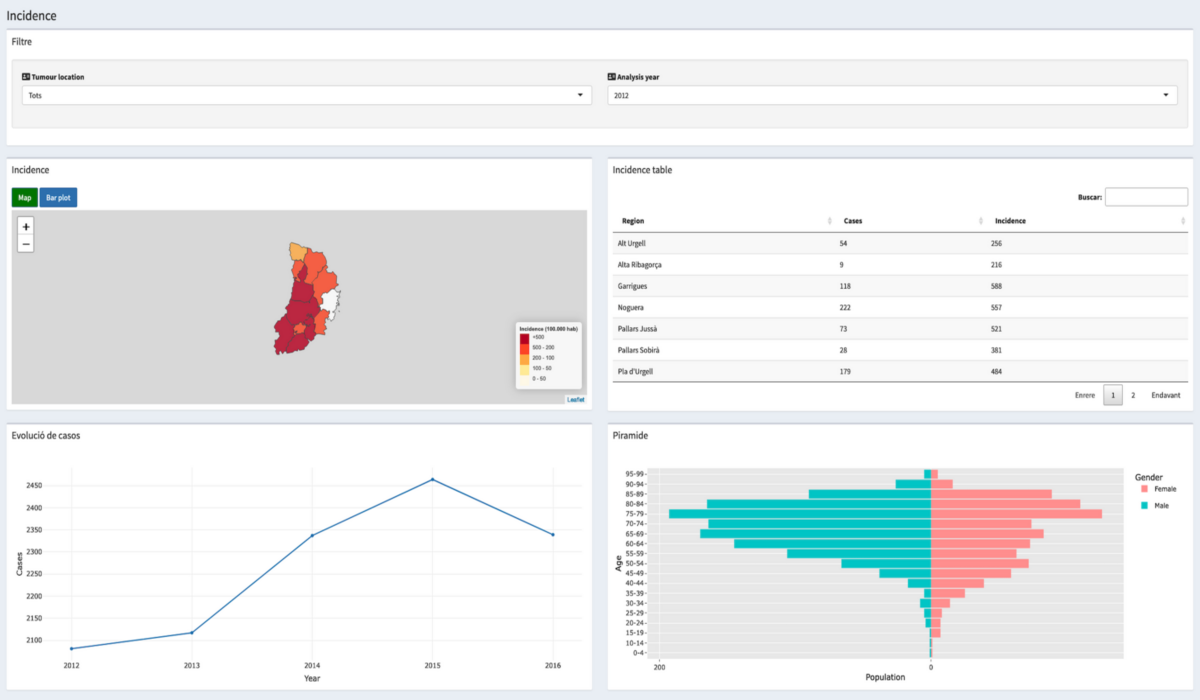
Specific incidence view.

Figure 2 shows a view for analyzing the incidence in the Lleida region. Specifically, it permits observation of diagnosed cases by year and cancer for specific subregions in Lleida, as the filter header represents. The view is also designed as a dashboard to enable user interaction. First, a heat map of the Lleida region is implemented. It shows the cancer incidence (per 100,000 habitants) for each area, where the color represents the incidence value. The view also offers analysis of this incidence in a bar plot (see the blue button in the map box). On the right, it shows a table with the number of cases and incidence for each area represented in the map information. These 2 elements are updated by year and the kind of cancer the user chooses in the filter. Below them, there is an evolution plot of the number of cancer cases registered. This plot is only recalculated when the user chooses a different cancer, and the year filter does not affect it. Finally, the age pyramid plot is represented, and it can be calculated by cancer and year.

### Cancer Risk Factors

**Figure 3:**
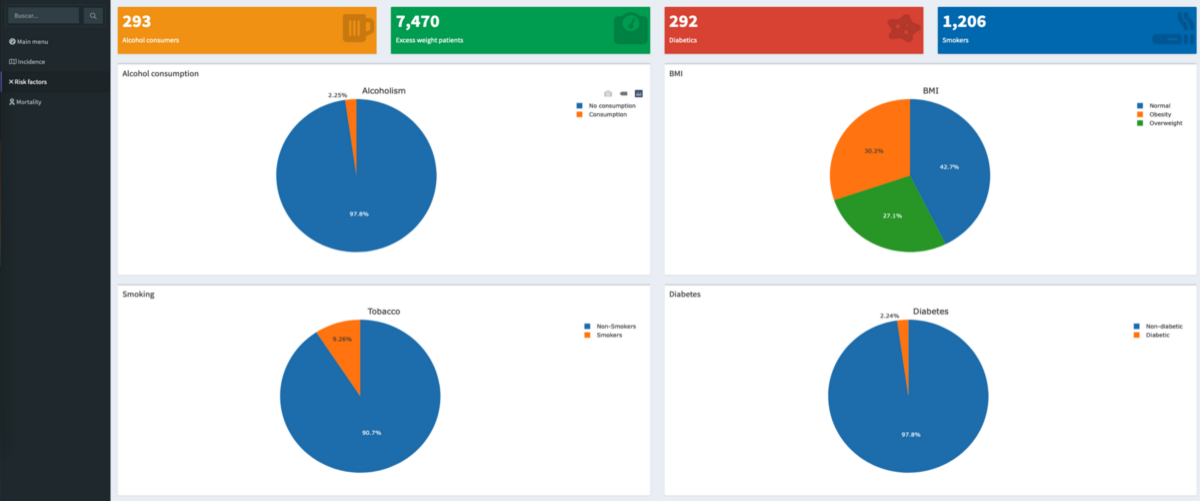
Risk factors view.

This view permits the risk factors’ impact on cancer patients to be analyzed. Figure 3 shows 4 value boxes with the number of cases for each risk factor. First, it shows the number of patients exposed to alcohol consumption before a cancer diagnosis. Next, the number of patients with excess weight (overweight or obese) and the number of patients diagnosed with diabetes before tumor registration are shown. Finally, the number of smokers among all those who were registered is shown. Below the value box, 4 pie charts were designed to compare the exposure to these risk factors. First, alcohol risk was represented, and only 2.2% (293/13,030) of the patients were exposed. On the right, body mass index was defined; overweight affected 27.1% (3532/13,030) of the patients, and obesity affected 30.2% (3938/13,030) of the patients. At the bottom, smoking was reported for 9.3% (1212/13,030) of patients, and diabetes was reported for 2.2% (292/13,030) of patients.

### Cancer Mortality

**Figure 4:**
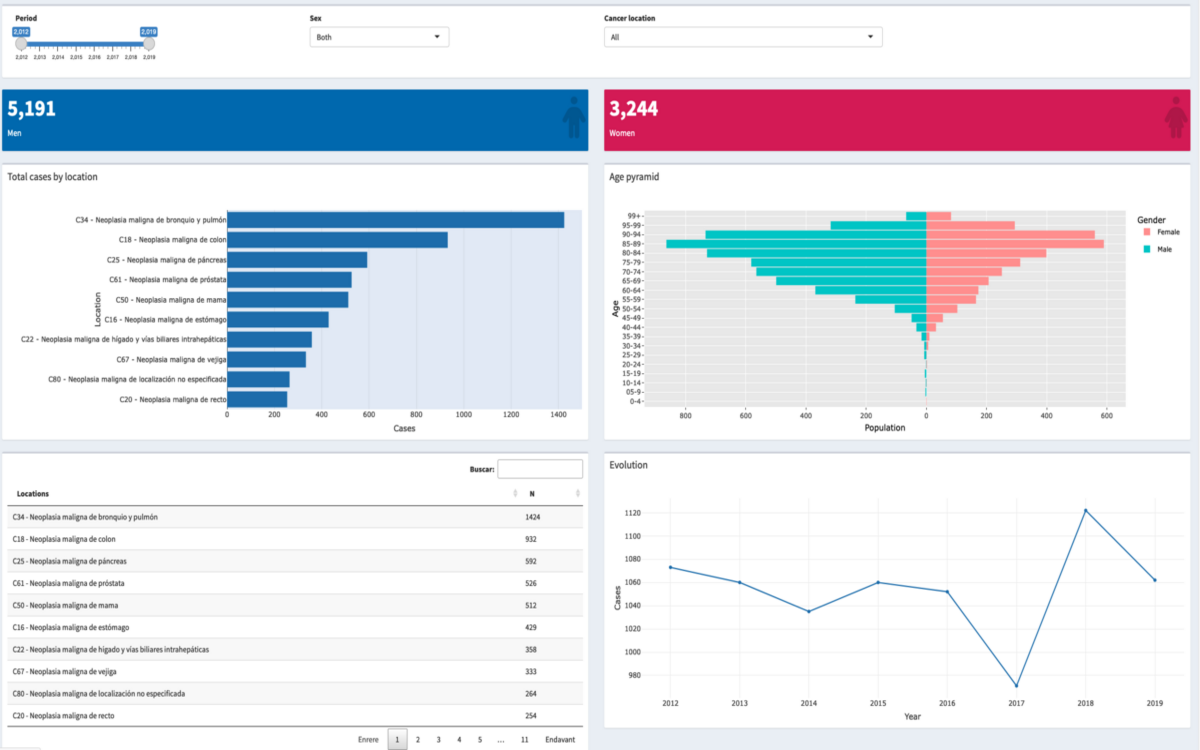
Mortality view.

The last implemented view shows an analysis of Lleida residents affected by tumors. In this case, the observed years were between 2012 and 2019 because the Mortality Register of Catalonia was already available for this time. Therefore, as Figure 4 shows, the filter box enables filtering by a period of years or by only 1 year. It permits showing the information by only men or women and by specific tumor location. Below the filter box, the user sees 2 value boxes representing the number of men and women who passed away among the chosen years and by tumor location. When a specific gender is selected, the other is hidden, making visible the value box chosen in the filter.

This view also contains 4 figures, 3 plots, and 1 table. At the top left, there is a horizontal bar plot representing the 10 tumors with the most cases of mortality. It is recalculated by the period and gender chosen; the filtered cancer location does not affect it. On the right, an age pyramid plot analyzes the mortality in each age group by gender. This plot can also be recalculated by the period in years and by cancer location. At the bottom, a table has the tumor locations and the number of patients who passed away, sorted in descending order. The information is displayed by the chosen period of years and gender; the cancer location filter will not affect it. Finally, an evolution plot is calculated to analyze the increase or decrease in deaths for all locations or specific tumors. This plot is recalculated depending on the chosen year, gender, or tumor location.

### Customization

The research team designed the system for easy deployment. Therefore, the users only need to consider these items:

- Deploy the Mongo database by executing the docker-compose file. The system will download the Mongo image (if it is the first time it runs), build the Docker Container, and deploy the database. Finally, add the information to show in the dashboard web application.
- Download the web application project and specify the user and password in the config.js file. Next, execute the docker-compose file to build the containers for the API system and R Shiny application. The system will download the image to make these containers if it is the first time and then deploy the containers.

## Discussion

### Principal Findings

The research team designed and implemented a web application to rapidly analyze the cancer situation in the Lleida region. It contains information about the incidence of each cancer by subregion, related risk factors, and the cancer mortality registered in this region. The application can be used in computer and mobile browsers because it has been designed responsively. It has been implemented using open-source technologies such as Docker, MongoDB, NodeJS, and R Shiny, which permit easy deployment of cancer registries in other hospitals. The code is also free to download and can be deployed within 1 day.

Recently, new applications have been designed to facilitate the analysis of data sets. Some studies have suggested that the latest technologies can help to extract information and value of the data rapidly and obtain the results instantly in different contexts. Luz et al [27] designed an application called RadarR to analyze infection management. They described an accessible web application to analyze infection and antimicrobial stewardship information. Another study implemented a Shiny application for automatically coding text responses [28]. They offer an application in which users can add text to train a model to analyze this added information. For completely different information but with the same technologies, Möller et al [29] presented an R Shiny application for the visualization and extraction of phenological windows in Germany. As the literature shows, these kinds of applications are increasing for all themes as well as cancer. Miller and Shalhout [30] designed and implemented an application to generate anatomical visualizations of cancer lesions. They concluded that data visualizations of the characteristics of clinical tumors could help to understand the natural history of malignancies. Therefore, this interactive data visualization application could permit analysis of the tumor characteristics. Another R Shiny application related to cancer data was published by Zhang et al [31]. The researchers designed a platform to analyze cell line responses to an anticancer drug. They concluded that it helped researchers understand the response of tumor cell lines to 15 therapeutic agents. Finally, a similar platform was implemented by Xia et al [32]. This platform visualizes cancer risk factors and mortality [32]. They shared a data warehouse and R Shiny application to improve their understanding of spatial and temporal trends across the population served by the University of Kansas Cancer Center.

This system helped the research team rapidly analyze the cancer information and reach some conclusions about the data and the use of these technologies. Therefore, regarding cancer incidence, the analysis detected that the number of cases is higher in men than in women in all periods and years [33]. Regarding age, the average age was 67 years, considering both genders. Men aged 65 years to 79 years registered a significant number of cases. However, cases for women occurred more often between 65 years and 69 years of age and between 75 years and 84 years of age [34]. Additional observable information was that the most common were cancers of the colon, lung, breast, prostate, and bladder [33,34]. Finally, an evolution of the incidence in Lleida showed an increase in the cases until 2015. The specific cancer incidence view also gave important information about some regions in Lleida. We observed that some areas, considered more urban than rural, had a higher incidence of some kinds of cancer, such as colon or lung [35,36].

As the incidence showed, the risk factors view also provided the previous situation of patients with cancer. Regarding risky drinking, 2.2% of the patients diagnosed consumed high amounts of alcohol daily [37]. The same percentage, 2.2%, of patients had diabetes. However, smokers represented 9.3% of the patients, one of the highest risk factors related to cancer [38]. Finally, the percentage with excess weight was high (57.3%), and some studies have pointed out that excess weight is significantly associated with the risk of cancer [39]. These results, including the number of cases for each risk factor, were obtained by the implementation of this application, which also helps to understand the cancer situation better, as other research teams have done before [32,40].

The cancer mortality registry permitted us to analyze the severity and impact of this disease, considered the second cause of death globally [41]. As we showed previously, analysts need tools like our web application offers. The application indicated that more men than women died between 2012 and 2019 [42], which might be related to the number of observed cases of cancer diagnosed among men and women [33]. The application also permitted us to know that lung cancer was the most lethal cancer among men [43] and breast cancer was the most lethal cancer in women [44]. Regarding age, the age group of 85 years to 89 years registered the highest number of deaths in both genders. Finally, we observed a general decrease in cancer deaths until 2018, when the number of patients passing away increased significantly. In case a user wanted to analyze a specific cancer location, the web platform recalculates the plots and tables for this variable.

The application presents some strengths and limitations that should be noted. This kind of implementation increases the data’s potential and adds value to the cancer registries. It permits an analysis and comparison of cancer information trends in specific areas in real time and helps make decisions about public health and the impact of cancer. The risk factor situation among cancer patients suggests some associations between risk factors and cancer. The scalability of the technologies used helps to deploy them to other cancer registries. Regarding limitations, the map plot has to be adapted to the region where it is deployed. The inconsistency between the cancer registry and cancer mortality did not permit them to be merged and analyzed in depth. The codification of some risk factors suggested underdiagnosis. A future systematic link between the cancer registry and the primary care medical records could improve the registry of risk factors. Related to the software, R Shiny presented some restrictions and incompatibility with some new libraries even though they were supplied with others that are accepted and adapted perfectly. MongoDB, in the beginning, requires extra effort to understand how it works, which delayed other parts of the application.

### Conclusions

The web application discussed in this study offers an analytical model of population cancer information. In addition, the technologies used to build this system permit its deployment into other cancer registries. Although there are web applications based on similar technologies, none use population cancer registry data to show the cancer situation in a specific region.

The views presented in the platform show the incidence of cancer detected in a specific time and particular areas, allowing it to be filtered by such inputs as year, gender, and tumor location. It also shows the evolution of cancer in the years analyzed. In addition, it studies the impact of some risk factors among the patients in the registry. Finally, it permits users to explore cancer mortality and its evolution in the Lleida region, filtering by year, gender, and tumor location.

Regarding future work, the research team is designing new views to analyze cancer incidence and the impact of the second primary tumor in depth. They are also creating a new risk factor view to offer a filter to give the risk factors for specific gender and tumor locations and integrating treatment data, such as for radiotherapy and chemotherapy. Finally, new web views are being created to build machine learning algorithms, train models, and analyze the results.

## Abbreviations

API: application programming interface
CEIC: Committee of Ethics and Clinical Research of Lleida
CI5: Cancer Incidence in Five Continents
PBCR: population-based cancer registry

## Data Availability

The data set is available from the corresponding author upon reasonable request.
